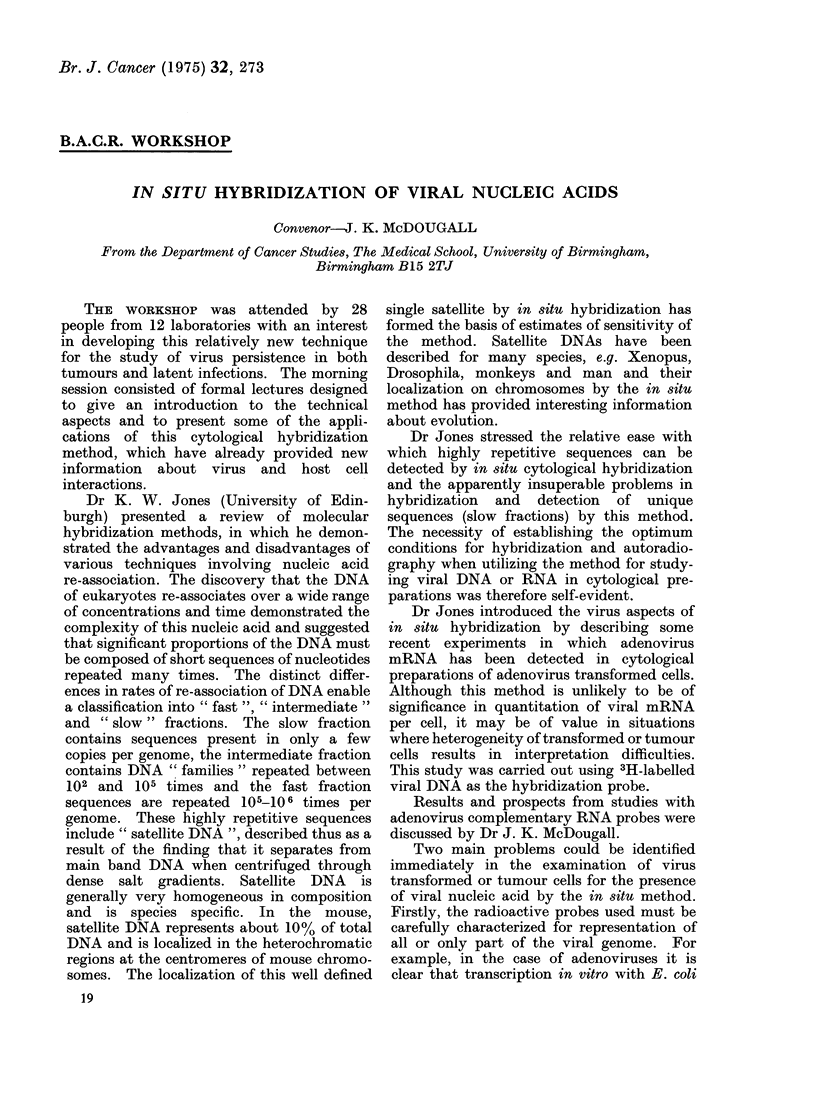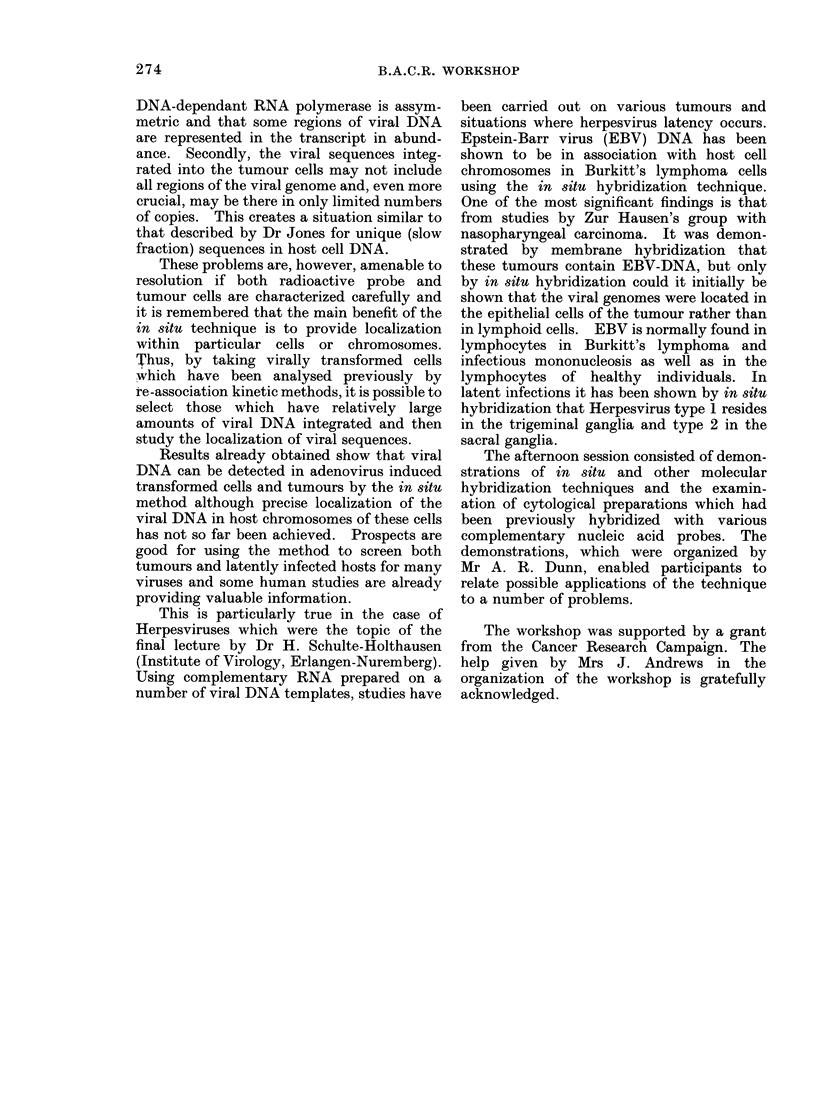# In Situ Hybridization of Viral Nucleic Acids

**Published:** 1975-08

**Authors:** 


*Br. J. Cancer* (1975) **32**, 273

### B.A.C.R. WORKSHOP

### *IN SITU* HYBRIDIZATION OF VIRAL NUCLEIC ACIDS

*Convenor*—J. K. McDOUGALL

*From the Department of Cancer Studies, The Medical School, University of Birmingham, Birmingham B15 2TJ*

THE WORKSHOP was attended by 28 people from 12 laboratories with an interest in developing this relatively new technique for the study of virus persistence in both tumours and latent infections. The morning session consisted of formal lectures designed to give an introduction to the technical aspects and to present some of the applications of this cytological hybridization method, which have already provided new information about virus and host cell interactions.

Dr K. W. Jones (University of Edinburgh) presented a review of molecular hybridization methods, in which he demonstrated the advantages and disadvantages of various techniques involving nucleic acid re-association. The discovery that the DNA of eukaryotes re-associates over a wide range of concentrations and time demonstrated the complexity of this nucleic acid and suggested that significant proportions of the DNA must be composed of short sequences of nucleotides repeated many times. The distinct differences in rates of re-association of DNA enable a classification into " fast ", " intermediate " and " slow " fractions. The slow fraction contains sequences present in only a few copies per genome, the intermediate fraction contains DNA " families " repeated between $10^2$ and $10^5$ times and the fast fraction sequences are repeated $10^5$–$10^6$ times per genome. These highly repetitive sequences include " satellite DNA ", described thus as a result of the finding that it separates from main band DNA when centrifuged through dense salt gradients. Satellite DNA is generally very homogeneous in composition and is species specific. In the mouse, satellite DNA represents about 10% of total DNA and is localized in the heterochromatic regions at the centromeres of mouse chromosomes. The localization of this well defined single satellite by *in situ* hybridization has formed the basis of estimates of sensitivity of the method. Satellite DNAs have been described for many species, *e.g.* Xenopus, Drosophila, monkeys and man and their localization on chromosomes by the *in situ* method has provided interesting information about evolution.

Dr Jones stressed the relative ease with which highly repetitive sequences can be detected by *in situ* cytological hybridization and the apparently insuperable problems in hybridization and detection of unique sequences (slow fractions) by this method. The necessity of establishing the optimum conditions for hybridization and autoradiography when utilizing the method for studying viral DNA or RNA in cytological preparations was therefore self-evident.

Dr Jones introduced the virus aspects of *in situ* hybridization by describing some recent experiments in which adenovirus mRNA has been detected in cytological preparations of adenovirus transformed cells. Although this method is unlikely to be of significance in quantitation of viral mRNA per cell, it may be of value in situations where heterogeneity of transformed or tumour cells results in interpretation difficulties. This study was carried out using $^3$H-labelled viral DNA as the hybridization probe.

Results and prospects from studies with adenovirus complementary RNA probes were discussed by Dr J. K. McDougall.

Two main problems could be identified immediately in the examination of virus transformed or tumour cells for the presence of viral nucleic acid by the *in situ* method. Firstly, the radioactive probes used must be carefully characterized for representation of all or only part of the viral genome. For example, in the case of adenoviruses it is clear that transcription *in vitro* with *E. coli*

DNA-dependant RNA polymerase is assymmetric and that some regions of viral DNA are represented in the transcript in abundance. Secondly, the viral sequences integrated into the tumour cells may not include all regions of the viral genome and, even more crucial, may be there in only limited numbers of copies. This creates a situation similar to that described by Dr Jones for unique (slow fraction) sequences in host cell DNA.

These problems are, however, amenable to resolution if both radioactive probe and tumour cells are characterized carefully and it is remembered that the main benefit of the *in situ* technique is to provide localization within particular cells or chromosomes. Thus, by taking virally transformed cells which have been analysed previously by re-association kinetic methods, it is possible to select those which have relatively large amounts of viral DNA integrated and then study the localization of viral sequences.

Results already obtained show that viral DNA can be detected in adenovirus induced transformed cells and tumours by the *in situ* method although precise localization of the viral DNA in host chromosomes of these cells has not so far been achieved. Prospects are good for using the method to screen both tumours and latently infected hosts for many viruses and some human studies are already providing valuable information.

This is particularly true in the case of Herpesviruses which were the topic of the final lecture by Dr H. Schulte-Holthausen (Institute of Virology, Erlangen-Nuremberg). Using complementary RNA prepared on a number of viral DNA templates, studies have been carried out on various tumours and situations where herpesvirus latency occurs. Epstein-Barr virus (EBV) DNA has been shown to be in association with host cell chromosomes in Burkitt's lymphoma cells using the *in situ* hybridization technique. One of the most significant findings is that from studies by Zur Hausen's group with nasopharyngeal carcinoma. It was demonstrated by membrane hybridization that these tumours contain EBV-DNA, but only by *in situ* hybridization could it initially be shown that the viral genomes were located in the epithelial cells of the tumour rather than in lymphoid cells. EBV is normally found in lymphocytes in Burkitt's lymphoma and infectious mononucleosis as well as in the lymphocytes of healthy individuals. In latent infections it has been shown by *in situ* hybridization that Herpesvirus type 1 resides in the trigeminal ganglia and type 2 in the sacral ganglia.

The afternoon session consisted of demonstrations of *in situ* and other molecular hybridization techniques and the examination of cytological preparations which had been previously hybridized with various complementary nucleic acid probes. The demonstrations, which were organized by Mr A. R. Dunn, enabled participants to relate possible applications of the technique to a number of problems.

The workshop was supported by a grant from the Cancer Research Campaign. The help given by Mrs J. Andrews in the organization of the workshop is gratefully acknowledged.